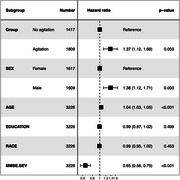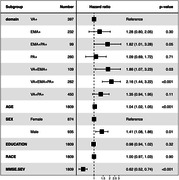# Agitation and mortality in dementia: application of the syndromic International Psychogeriatric Association criteria‐based approach to survival analyses

**DOI:** 10.1002/alz.093406

**Published:** 2025-01-03

**Authors:** Zahinoor Ismail, Zahra Hosseinpour, Chenhui Hao, Zahra Goodarzi, Eric E. Smith, Dallas Seitz

**Affiliations:** ^1^ Hotchkiss Brain Institute, University of Calgary, Calgary, AB Canada; ^2^ University of Calgary, Calgary, AB Canada; ^3^ Department of Psychiatry, University of Calgary, Calgary, AB Canada

## Abstract

**Background:**

Agitation in dementia is associated with poor outcomes, including mortality. However, the lack of a consensus definition of agitation has hampered research, historically driven by variable approaches to symptom identification. Accordingly, prevalence estimates range widely (5%‐88%) and interventions are hard to compare meaningfully. The International Psychogeriatric Association (IPA) consensus definition advanced the field from a symptomatic to a syndromic criteria‐based approach. As per the validated IPA criteria, agitation behaviors can manifest as verbal aggression (VA), physical aggression (PA), and/or excessive motor activity (EMA). We determined the three‐year mortality rate for syndromic agitation and each of the three IPA agitation domains. We hypothesized 1) higher mortality for those with agitation; and 2) PA would have the highest mortality of the three domains.

**Method:**

National Alzheimer’s Coordinating Center (NACC) participants (n = 3226), with baseline dementia diagnosis were included. Neuropsychiatric Inventory Questionnaire (NPI‐Q) items identified IPA domains as follows: VA (irritability); PA (agitation/aggression); and EMA (aberrant motor activity). Agitation+ status was based on item scores ≥1. Kaplan‐Meier survival curves were used to visualize and compare group trajectories. Cox proportional hazards regressions modelled: 1) three‐year mortality for Agitation±; and 2) three‐year mortality for combinations of agitation domains relative to VA alone, adjusted for age, sex, race, education, and MMSE. To address potential confounding, propensity scores were applied using inverse probability treatment weighting. Sex differences in outcomes across exposure groups were assessed.

**Result:**

Participants (age 74.5±9.7 years, 51% female) who were Agitation+ had a 1.37‐fold higher mortality rate (CI:1.12–1.69, p<0.001) relative to Agitation‐ (Figure 1). Higher agitation‐related mortality was seen in males (HR = 1.52, CI:1.18‐1.97, p = 0.001) and females (HR = 1.51, CI:1.1‐2.06), p<0.001); between‐sex HRs did not differ (multiplicative interaction test HR = 0.97, CI:0.8‐1.17, p = 0.96). Domain analyses revealed 1) generally higher hazards when >1 agitation domain was observed; and 2) higher hazard for mortality for EMA than PA in reference to VA (Figure 2).

**Conclusion:**

This study provides further validation of the IPA Agitation criteria and the syndromic approach to agitation in dementia. Previously underappreciated relative to PA, and contrary to our hypothesis, EMA emerged as an important IPA agitation domain, having the greatest mortality risk.